# 3D artificial round section micro-vessels to investigate endothelial cells under physiological flow conditions

**DOI:** 10.1038/s41598-018-24273-7

**Published:** 2018-04-12

**Authors:** Riccardo Sfriso, Shengye Zhang, Colette Andrea Bichsel, Oliver Steck, Alain Despont, Olivier Thierry Guenat, Robert Rieben

**Affiliations:** 1 Department for BioMedical Research, University of Bern, Bern, Switzerland; 2 Graduate School for Cellular and Biomedical Sciences, University of Bern, Bern, Switzerland; 3 First Affiliated Hospital of Zhengzhou University, Zhengzhou, China; 4 Vascular Biology Program, Boston Children’s Hospital and Harvard Medical School, Boston, MA USA; 5 ARTORG Center for Biomedical Engineering Research, University of Bern, Bern, Switzerland

## Abstract

In the context of xenotransplantation, in ischemia/reperfusion injury as well as in cardiovascular research, the study of the fascinating interplay between endothelial cells (EC) and the plasma cascade systems often requires *in vitro* models. Blood vessels are hardly reproducible with standard flat-bed culture systems and flow-plate assays are limited in their low surface-to-volume ratio which impedes the study of the anticoagulant properties of the endothelial cells. According to the 3R regulations (reduce, replace and refine animal experimentation) we developed a closed circuit microfluidic *in vitro* system in which endothelial cells are cultured in 3D round section microchannels and subjected to physiological, pulsatile flow. In this study, a 3D monolayer of porcine aortic EC was perfused with human serum to mimic a xenotransplantation setting. Complement as well as EC activation was assessed in the presence or absence of complement inhibitors showing the versatility of the model for drug testing. Complement activation products as well as E-selectin expression were detected and visualized *in situ* by high resolution confocal microscopy. Furthermore, porcine pro-inflammatory cytokines as well as soluble complement components in the recirculating fluid phase were detected after human serum perfusion providing a better overview of the artificial vascular environment.

## Introduction

Endothelial cell (EC) activation plays an important role in the pathophysiology of ischemia/reperfusion injury, sepsis, vascular rejection of transplanted organs, and other diseases linked to the vascular system. In transplantation, the vascular endothelium of the donor organ is the first tissue to come in contact with the blood of the recipient. If pre-formed anti-donor antibodies are present in the recipient’s blood, an immediate activation of the donor endothelium occurs due to antibody binding followed by activation of the complement system. This is for example the case in blood group ABO-incompatible transplantations, recipients sensitized to donor HLA antigens, and in experimental pig-to-primate xenotransplantation^1^. EC activation in turn triggers the coagulation cascade and leads to the clinical picture of hyperacute or acute vascular rejection^2,3^. Xenotransplantation experiments in animal models have been carried out extensively to investigate mechanisms of EC activation^4–6^, but also *ex vivo* perfusions of porcine organs with human blood, plasma or serum have been used for this purpose^7–9^. In order to reduce – in accordance with the 3R principles – the number of animals used for investigation of EC activation in hyperacute and acute vascular rejection, we developed an *in vitro* system to grow and investigate EC under physiological, pulsatile flow conditions, simulating shear stress as occurring in small to medium sized arteries. Furthermore, the system provides additional scientific advantages over *in vivo* models such as a reduced amount of drugs needed for the experiments, better controlled and lower variability, as well as the possibility to scale-up as a high-throughput system capable of parallel investigation of dozens or even more parameters like drugs or genetic modifications of EC.

In standard 2D cell culture the amount of serum, plasma or whole blood in contact with EC grown on the bottom of the wells is small and may often be the limiting factor for activation or cytotoxicity of EC *in vitro*: in a typical experiment using 96-well microtiter plates, the ratio of fluid volume to EC surface is only 0.2 ml/cm^2^ (100 µl per well with a bottom surface of 0.5 cm^2^). This ratio is much lower than in a physiological situation in which blood circulates through vessels and where ratios from 1.3 ml/cm^2^ (in the aorta) up to 5000 ml/cm^2^ (in capillaries) are reached. Using *in vitro* systems based on 3D culture of EC on the inner surface of ‘artificial blood vessels’ and perfusion with a physiological flow the *in vivo* ratio of fluid volume to EC surface can be reached making it possible to exploit the natural anticoagulant properties of EC^10^.

Over the last decade, microfluidic technologies have been developed, and commercial systems have been made available in which cells can be cultured under flow using convenient slide- or microtiter plate-based setups^11,12^. These systems are normally used to grow EC two-dimensionally, on the bottom of a rectangular shaped micro channel. Such systems have for example been used to assess the effect of complement inhibition on thrombus formation in a xenotransplantation setting^13,14^. Also 3D growth of EC has been reported on the inner surface of rectangular channels^15,16^. However, the geometry of these rectangular microfluidic channels poorly replicates the shape of the microvasculature, in particular in terms of shear stress. In order to fabricate circular microchannels, different technologies have been reported such as a combination of mechanical micromilling and soft lithography, or introducing a pressurized air stream into liquid uncured PDMS filled microchannels^17,18^. Most often, however, those “circular cross-sections” were rather irregular, making it difficult to standardize the respective assays and reproduce experimental findings.

Based on the use of needles as molds published by Chrobak *et al*.^19^, we therefore produced microfluidic chips with evenly circular microchannels. We inserted the needles directly in polydimethylsiloxane (PDMS) in a petri dish and extract them after casting the gel. This results in an even, round inner diameter of the microchannels, which remains constant at 37 °C. The inner surface of the microchannels is then chemically modified and functionalized to adsorb extracellular matrix proteins and allow cell attachment.

Existing microfluidic models often use syringe pumps kept outside the incubator with consequent temperature changing of the perfusate which might influence the behavior of the sensitive EC. The model presented here involves the use of a peristaltic pump and reservoir tubes which can be kept inside the incubator at 37 °C avoiding temperature changes of the medium while perfusing the cells. Recirculation is an interesting feature of the system as it allows for cell-cell communication through soluble messenger molecules such as cytokines, chemokines as well as amplification of plasma cascade systems.

In the present study porcine EC grown under physiological shear stress were perfused with normal human serum (NHS) as a source of xenoreactive natural antibodies and complement under physiological flow conditions in the context of xenotransplantation.

## Results

### Endothelial cell characterization

**Figure 1:**
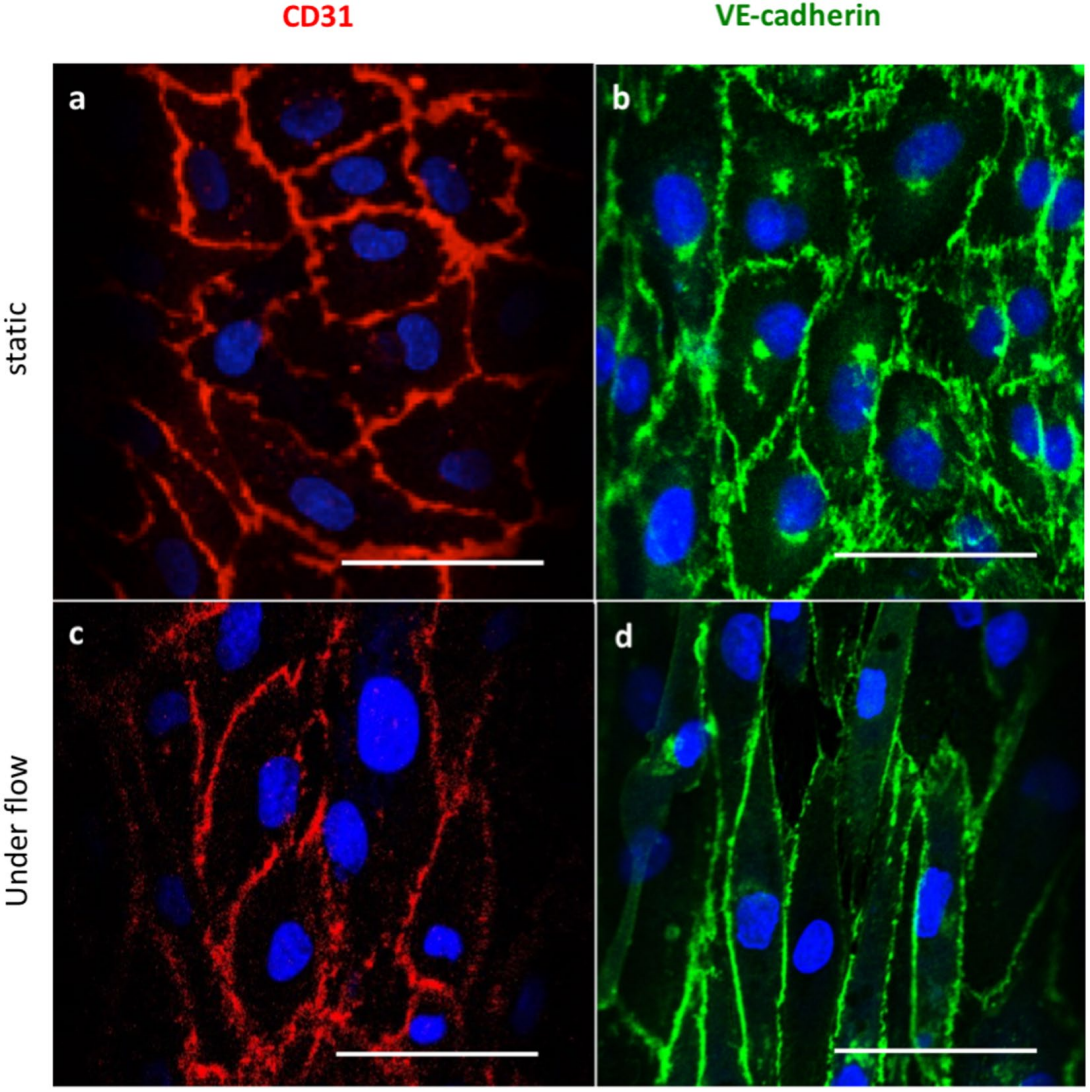
EC characterization and overview of cell distribution in microfluidic channels both under static and flow conditions. EC characterization by expression of CD31 and VE-cadherin. (**a**–**b**) Representative images for CD31 and VE-cadherin expression under static conditions; (**c**–**d**) representative images for CD31 and VE-cadherin expression under flow conditions. Scale bars represent 50 µm.

To confirm that EC isolated from porcine aortas still expressed typical endothelial markers when cultured in microfluidic channels, staining for CD31 and VE-cadherin was performed by immunofluorescence (IF). All of these markers were expressed on PAEC after culturing in the 3D microfluidic system under both static and flow conditions, demonstrating successful PAEC culture in the microfluidic channels (Fig. 1). However, expression of the respective markers was different depending on flow conditions. In cells cultured under static conditions, CD31 and VE-cadherin were expressed in arbitrary patterns, whereas CD31 and VE-cadherin were aligned with the direction of the pulsatile flow when the cells were cultured for 2 days at 10 dyn/cm^2^. This indicates that the expression of these endothelial cell markers is affected by shear stress-dependent mechanotransduction^20^.

### Cell morphology, alignment, and distribution along the direction of pulsatile flow

**Figure 2:**
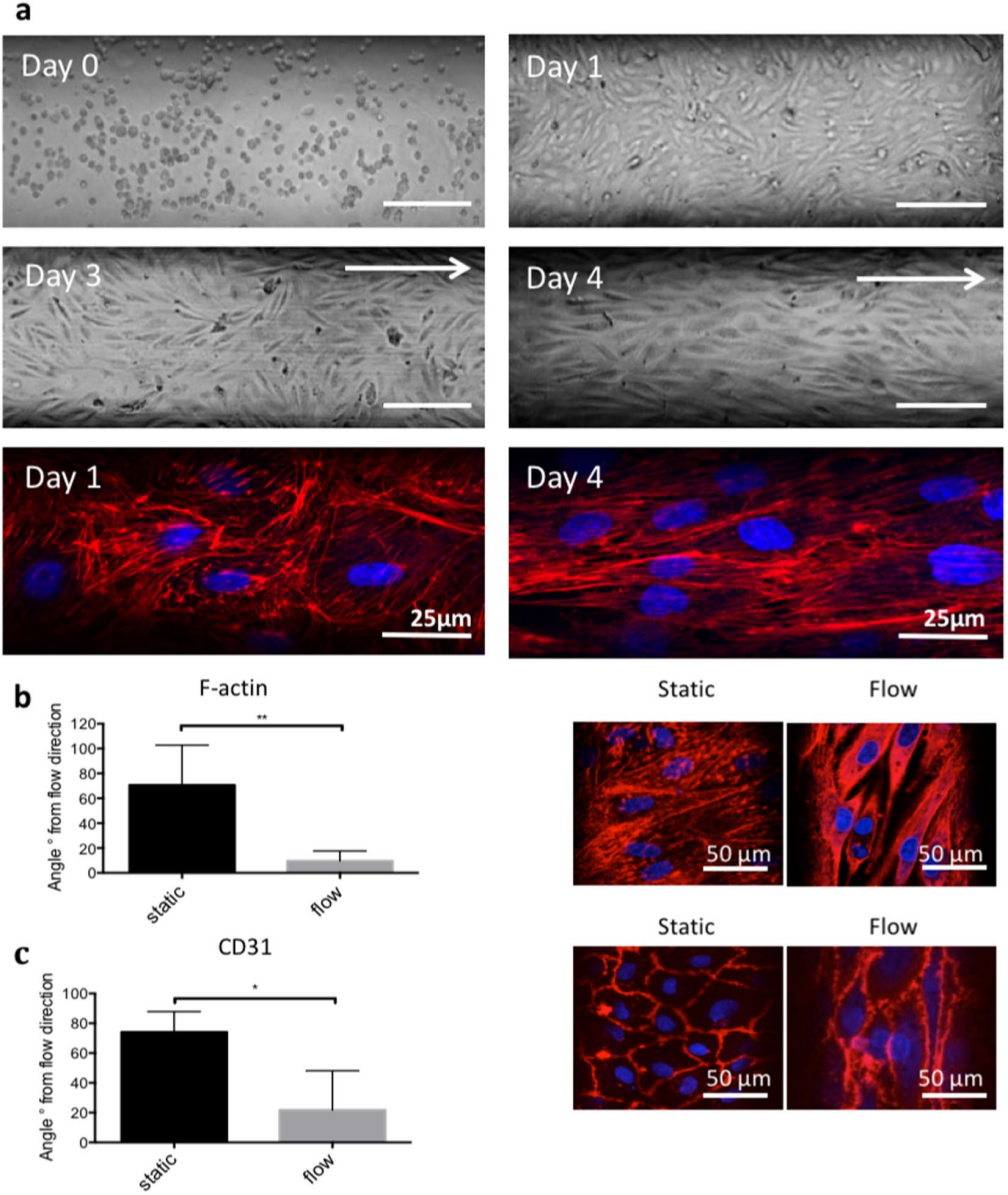
Cell morphology and quantification of cell alignment. (**a**) Cell morphology over time. (a) day 0, cells are randomly distributed immediately after seeding; (**b**) day 1, cells attach and elongate under static conditions; (**c**) day 3, cells start to become aligned under flow for one day; (**d**) day 4, most of the cells are aligned under flow for two days. Arrows indicate the direction of pulsatile flow in the microfluidic channels. (**e**) F-actin staining of PAEC in static conditions and (**f**) under flow. If not specified scale bar represents 100 µm. **(b**,**c**) Quantification of cell alignment to the x-axis of the microfluidic channels by immunofluorescence staining for the cytoskeleton protein F-actin and CD31, respectively. On the left panel, column graphs of the average cell angle in degrees to the x-axis are shown under static and pulsatile flow conditions (mean values ± SD, p-value: * < 0.05, ** < 0.01). Representative immunofluorescence images are shown on the right panel (a-b). Arrows show the flow direction. Scale bar represents 50 µm.

Cells started to attach to the inner surface of the microchannels 1 h after seeding. They then became elongated and a confluent EC monolayer was formed on day 1. When a pulsatile flow was applied, cells started to align with the flow over time. After 2 days of pulsatile flow, cells were completely aligned as shown by bright field microscopy pictures and F-actin staining at days 2 and 4 (Fig. 2a). Cell alignment in the direction of flow was assessed by staining of the cytoskeleton protein F-actin as well as CD31. For F-actin, after 2 days of pulsatile flow, the average angle of the cells with respect to the flow direction of the microchannels was 9.6 ± 8.1°, which was significantly smaller than under static conditions (70.7 ± 32.1°, p = 0.007). For CD31, the respective values were 21.8 ± 26.3° and 74.2 ± 13.7°, respectively, p = 0.047 (Fig. 2b,c).

This cell alignment was described earlier in microfluidic studies and is supposed to be due to mechanically affected distribution of cytoskeleton proteins as soon as exposure to shear stress occurs, which is induced by pulsatile perfusion with cell culture medium^21,22^.

**Figure 3:**
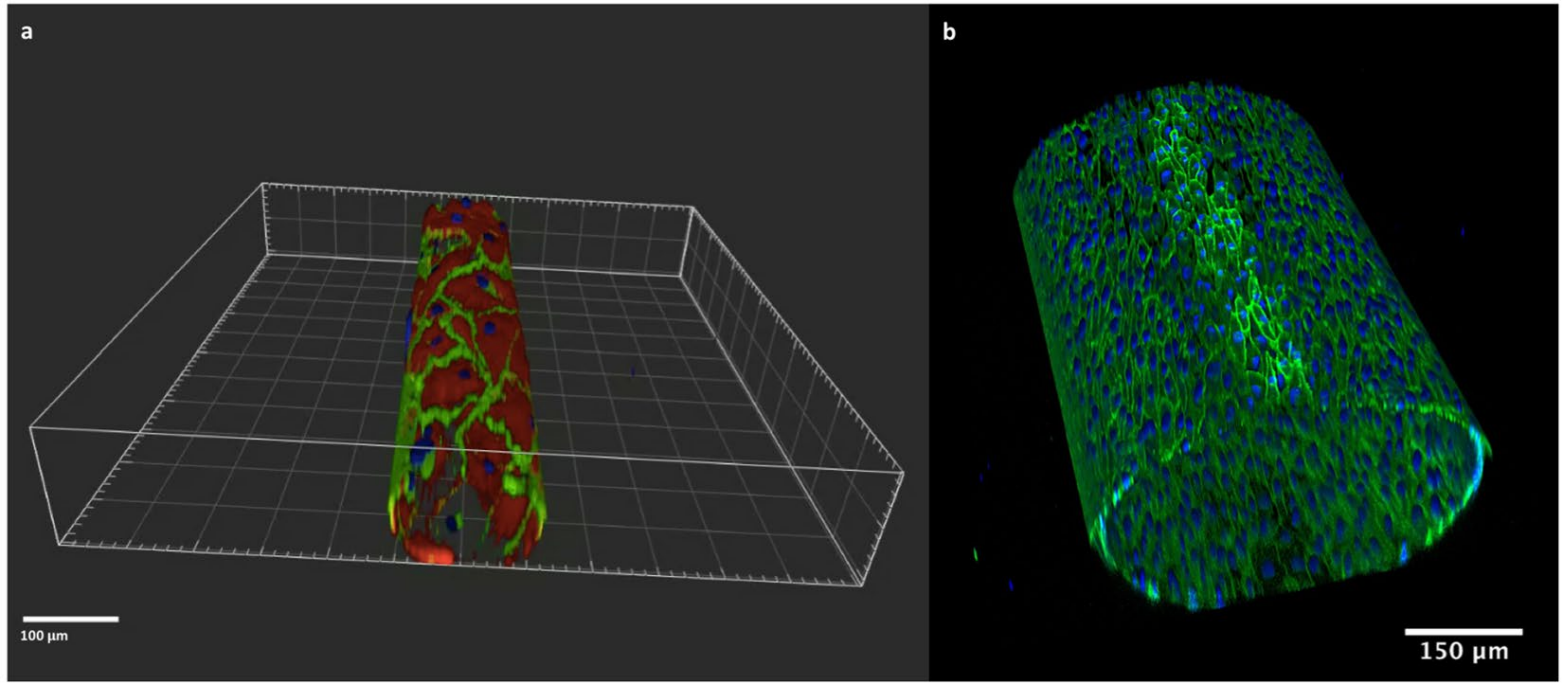
Confocal images of EC coated microchannels. (**a**) 3D rendering of the 100 µm round section channel. EC monolayer was stained for VE-cadherin (green) and F-Actin (red). Nuclei were stained with DAPI (blue). (**b**) 3D z-stack of the 550 µm round section channel. EC monolayer was stained for VE-cadherin (green). Nuclei were stained with DAPI (blue).

In our microfluidic system, the formation of an EC monolayer on the whole inner surface of the microchannels was assessed by IF and confocal microscopy. VE-cadherin staining showed elongated EC covering the entire microchannel and forming a typical monolayer on both 550 µm and 100 µm channel diameters (double staining with F-actin in 100 µm channels, Fig. 3a and supplementary movie 1). This observed staining pattern mimics the *in vivo* impression of a small artery as shown in 3D rendering views in Fig. 3a and b. In contrast to the *in vivo* situation, biological phenomena on a molecular or cellular level could be directly observed in our microfluidic assay by real time *in vivo* cell imaging even though the data presented here were obtained at the end of the experiments only. High resolution confocal laser scanning microscopy as well as spinning disk microscopy for high-speed acquisition of pictures can be used and provide detailed insights into biological mechanisms.

### Complement activation in a xenotransplantation setting

**Figure 4:**
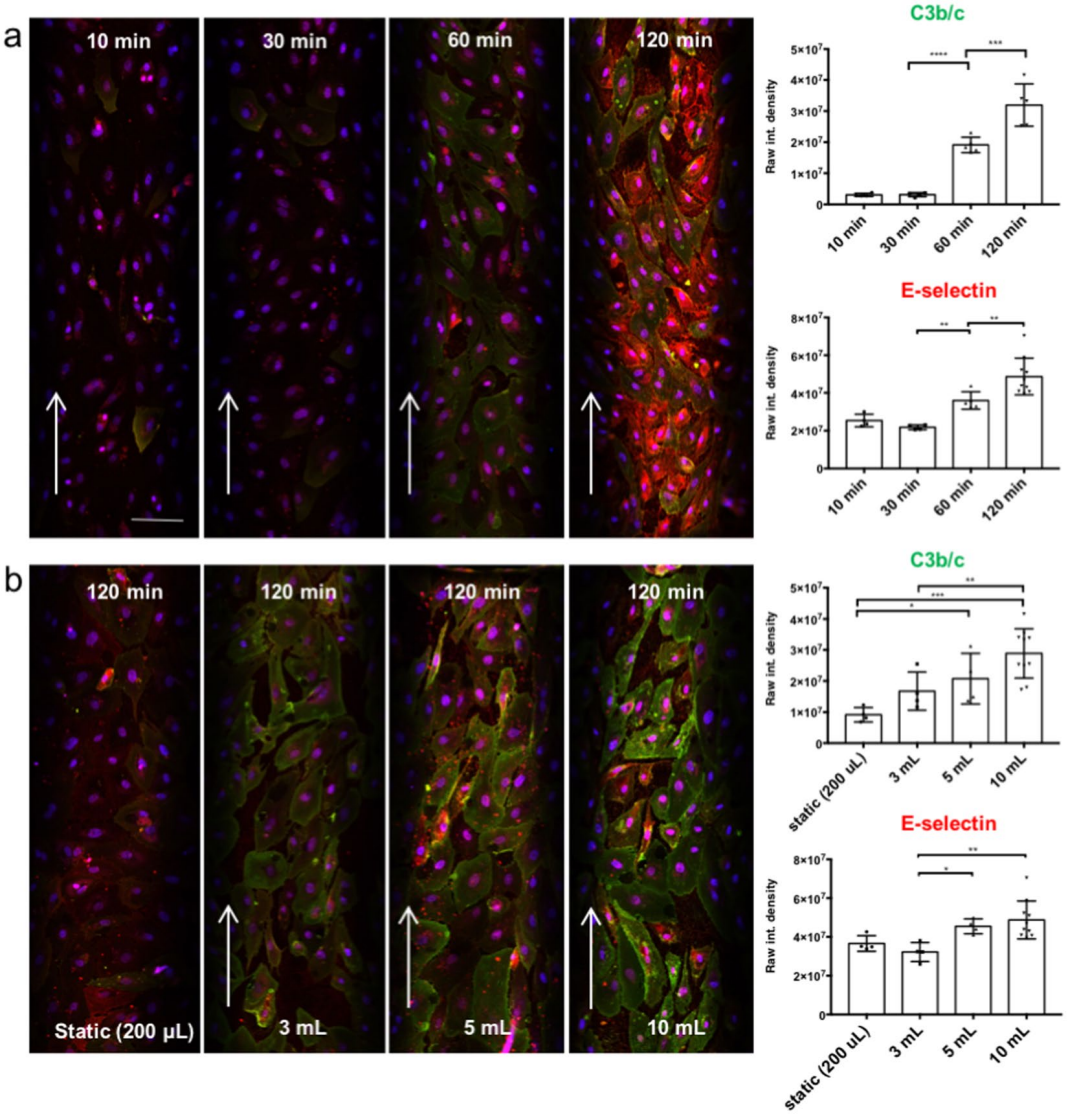
Impact of incubation time on EC activation and complement deposition. Confocal pictures of EC coated microchannels. (**a**) Perfusion with 10 ml of 1:10 diluted NHS, recirculating at 600 µl/min for 10, 30, 60, 120 min. (**b**) Incubation for 120 min with different volumes of 1:10 diluted NHS: 200 µl (static conditions), 3 ml, 5 ml, and 10 ml (all recirculating at 600 µl/min). Quantification of C3b/c deposition (green) and E-selectin expression (red) was done by immunofluorescence. Nuclei were stained with DAPI (blue). Arrows show the flow direction. Shown are mean values ± SD with indication of statistically significant differences between the time points, n = 5, p-value: *p = 0.01, **p < 0.01, ***p = 0.0001, ****p < 0.0001). Sera from different donors with different blood groups were used as pool of at least 3 donors. Scale bar represents 100 µm.

After establishment of our *in vitro* model, we aimed to reproduce complement activation as occurring in hyperacute or acute vascular rejection in a xenotransplantation setting^1^. We therefore perfused PAEC-microchannels with 1:10 diluted normal human serum. Dilution of the human serum is necessary to evaluate EC activation and complement deposition while minimizing cell loss as the immune reaction triggered by undiluted serum will result in rapid cell death and cell release from the channel surface. A monolayer of PAEC is essential to mimic an intact endothelium, therefore the intactness and the confluency of PAEC-coated microchannels were assessed before performing any experiment. However, since bovine collagen was used to coat the microchannels to allow a better cell adhesion and proliferation, binding of human antibodies was assessed by perfusing fibronectin/collagen coated microchannels with 1:10 NHS to verify the absence of xeno-reactions (Supplementary Fig. 1). The assessment of EC activation and complement deposition was performed by IF staining for E-selectin and C3b/c, respectively, after different serum incubation times: 10 min, 30 min, 60 min and 120 min. The results confirmed a time-dependently increased EC activation as shown by strong E-selectin expression and increased complement deposition as shown by C3b/c staining (Fig. 4a). Furthermore, another experiment was done by perfusing PAEC for 120 min with different volumes of 1:10 diluted NHS: 200 µl, 3 ml, 5 ml, 10 ml corresponding to 20 µl, 300 µl, 500 µl and 1 ml of undiluted NHS (Fig. 4b). The perfusion rate was kept constant at 600 µl/min except for the channels kept under static conditions which were filled with 200 µl of 1:10 diluted NHS. Significantly increased E-selectin expression and C3b/c deposition were observed when 5 ml and 10 ml of diluted NHS were used, corresponding to 500 µl and 1 ml of undiluted NHS, respectively. As control experiments both PAEC and human aortic endothelial cells (HAEC) were perfused with normal porcine serum (NPS) and NHS respectively (Supplementary Fig. 2). The data obtained support the idea that this microfluidic system, specifically optimized for the assessment and quantification of complement deposition thanks to the possibility to use relatively large volumes for perfusion of the artificial microvessels, is able to mimic the *in vivo* situation in which EC are continuously perfused with blood containing active proteins of the complement and coagulation cascade. Indeed, compared with standard chamber slides where the amount of serum is low (data not shown), our 3D microfluidic assay gave a better quantification of human immunoglobulin binding and complement deposition on porcine endothelial cells allowing to screen the protective role of transgenes.

**Figure 5:**
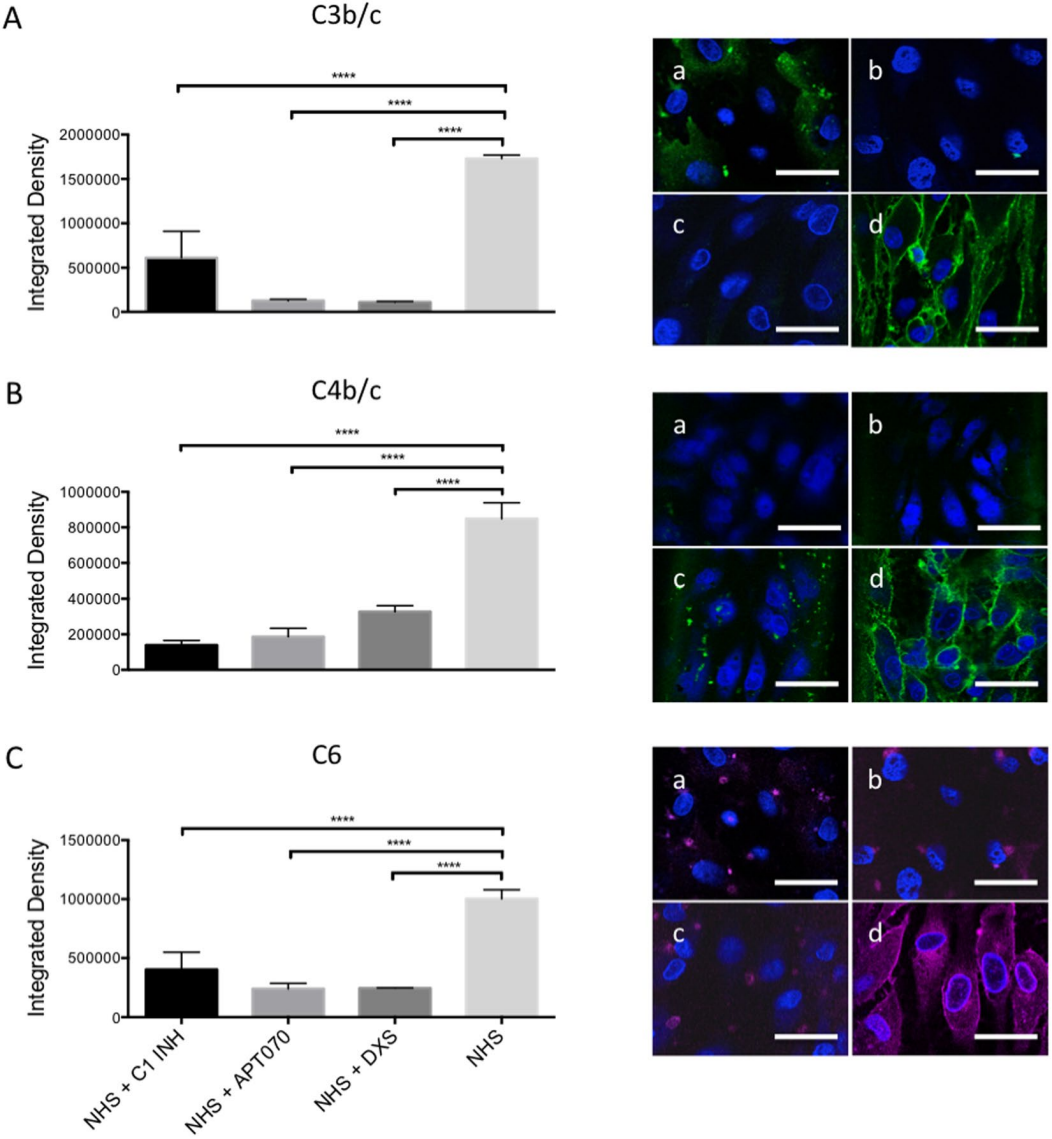
Deposition of C3b/c, C4b/c, and C6 on PAEC assessed by immunofluorescence after perfusion with NHS with or without complement inhibitors. Column graphs on the left panels show quantification of immunofluorescence staining for deposition of C3b/c (**A**), C4b/c (**B**) and C6 (**C**). Shown are mean values ± SD with indication of statistically significant differences between complement inhibitor groups and NHS alone. ****p < 0.0001, n = 5. Representative images are shown on the right panels. PAEC in microfluidic channels were perfused for 60 min with 1:10 NHS + C1 INH (**a**), 1:10 NHS + APT070 (**b**), 1:10 NHS + DXS (**c**), and 1:10 NHS alone (**d**). Sera from different donors with different blood groups were used as pool of 8 donors. Scale bar represents 50 µm.

An interesting application of our microfluidic system could be the screening of complement inhibitors or other drugs in general. Three known complement inhibitors were therefore tested in our model: C1 INH (10 IU/ml), APT070 (0.25 mg/ml), and DXS (0.3 mg/ml). C1 INH is a physiological, fluid phase inhibitor of complement and coagulation, acting mainly on the C1 complex, which initiates the classical pathway of complement activation^23^. APT070 is a recombinant derivative of the soluble complement receptor 1, regulating complement activation at the level of C4/C3^24^. DXS, finally, is a highly sulfated polyglucose and a member of the glycosaminoglycan family. It acts as an EC protectant and a complement inhibitor^25,26^. Activation of the complement cascade was confirmed by positive staining for C3b/c, C4b/c, and C6. As expected, all inhibitors blocked complement activation on the C4/C3 level and further downstream. Deposition of C3b/c, C4b/c, and C6 was significantly reduced by all of the used complement inhibitors compared to perfusion by NHS alone. The respective data are shown in Fig. 5, both quantitated as column graphs and as representative immunofluorescence images. Our results confirm earlier data on successful complement inhibition using C1 INH, APT070 and DXS^25,27,28^. Furthermore, the model could reproduce data obtained *ex vivo* in a pig lung xenotransplantation model by using the same amount of C1 INH (10 IU/ml) which was shown to effectively prolong the survival time of the xenoperfused organ by diminishing complement activation after perfusion with human blood^29^.

### Proinflammatory cytokines, growth factors and soluble complement activation markers in perfusate samples

**Figure 6:**
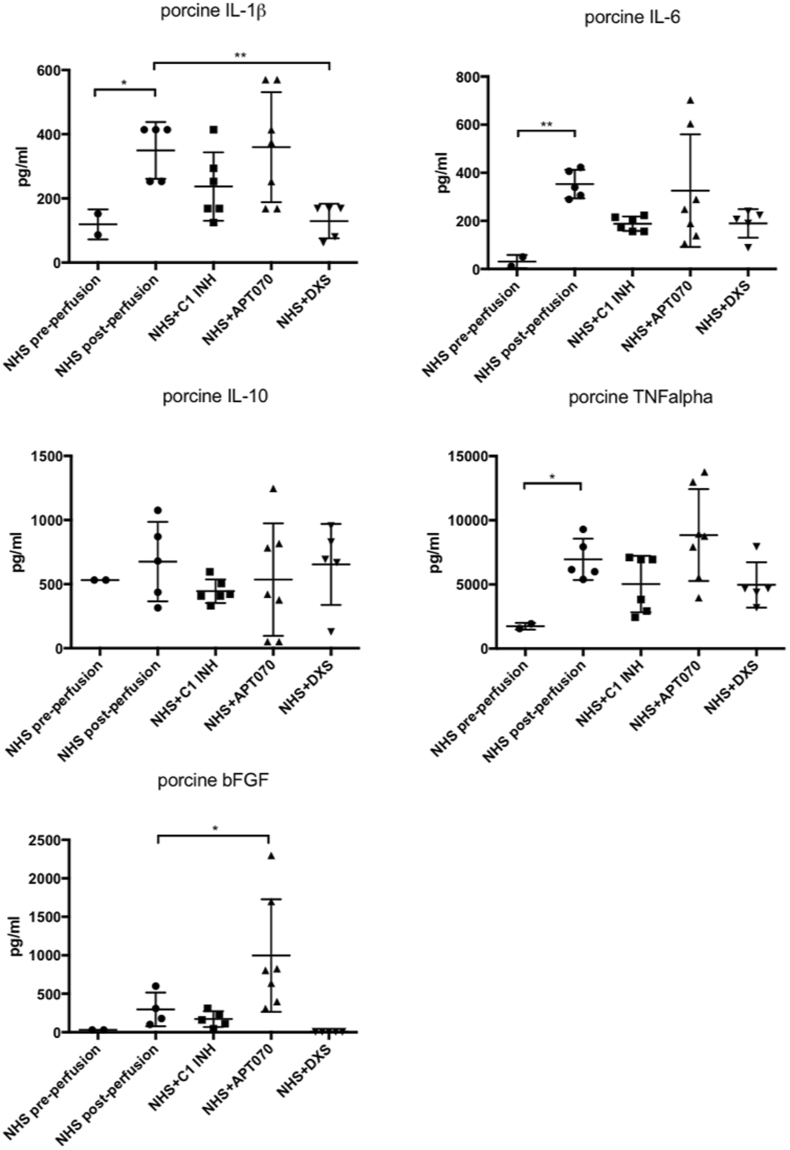
Concentrations of inflammatory cytokines/growth factors after perfusion with NHS with or without complement inhibitors. Porcine-specific cytokines [interleukin (IL)-6, IL-1β, IL-10, tumor necrosis factor alpha (TNF-α)] and basic fibroblast growth factor (bFGF) levels were measured by Bio-Plex analysis. Data are presented as scattered dot plot with mean values, error bars indicate standard deviations, NHS post-perfusion n = 5, NHS + C1INH n = 6, NHS + APT070 n = 7, NHS + DXS n = 5, p-values: *p < 0.05, **p < 0.01, ****p < 0.0001. Sera from different donors with different blood groups were used as pool of 8 donors.

**Figure 7:**
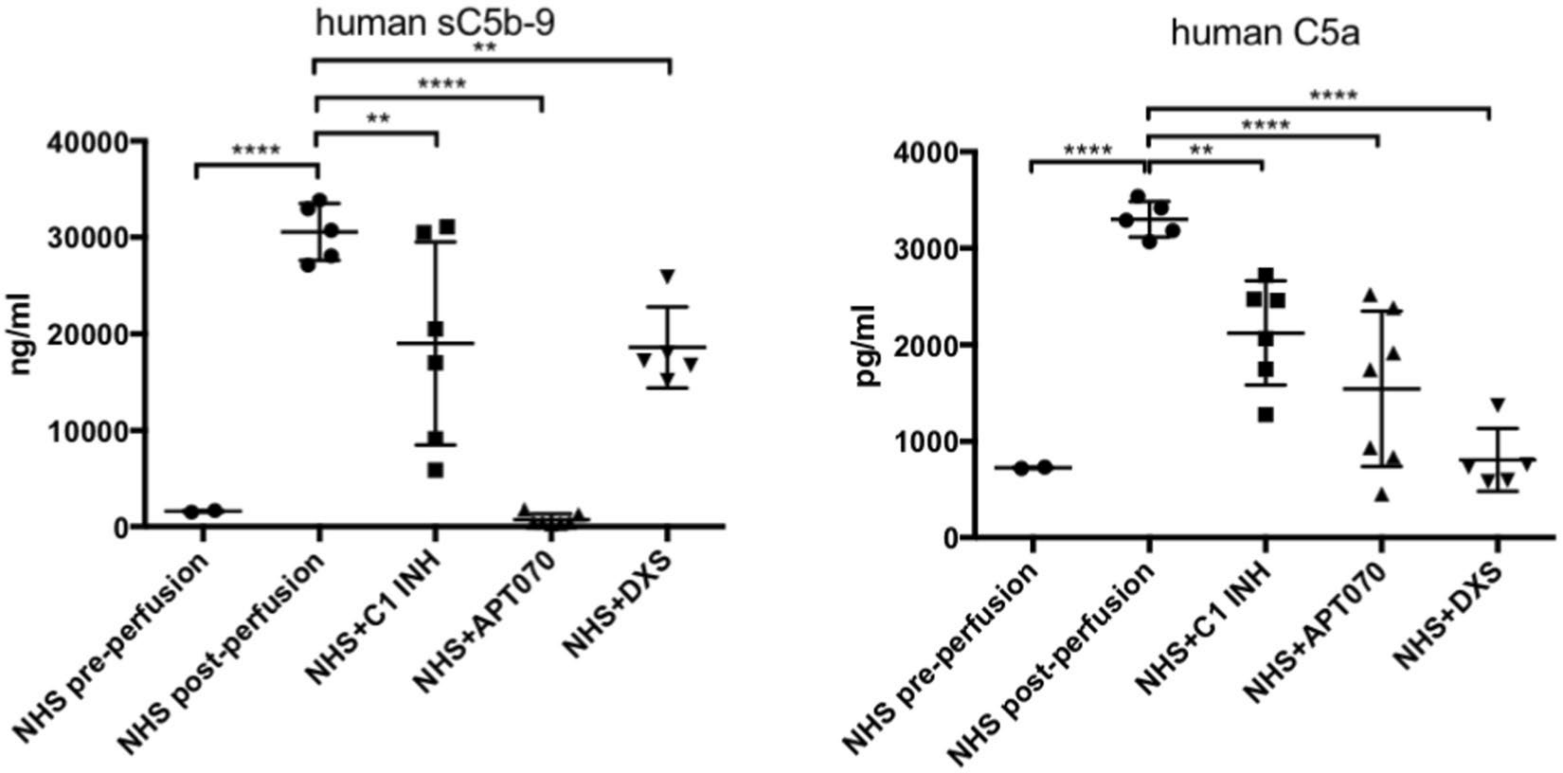
Concentrations of soluble complement markers after perfusion with NHS with or without complement inhibitors. Human soluble complement activation markers in the perfusate were detected by ELISA (human C5a) and Bio-Plex [human soluble (s)C5b-9]. Data are presented as scattered dot plot with mean values, error bars indicate standard deviations, NHS post-perfusion n = 5, NHS + C1INH n = 6, NHS + APT070 n = 7, NHS + DXS n = 5, p-values: **p < 0.01, ****p < 0.0001. Sera from different donors with different blood groups were used as pool of 8 donors.

Perfusate samples were collected and analyzed for the presence of porcine specific inflammatory cytokines, growth factors and soluble complement components. The assay specifically detects cytokines produced by porcine endothelial cells after being stimulated with NHS, with the exception of bFGF and sC5b-9 for which also the human proteins are detected. Analysis of NHS pre-perfusion as well as normal pig serum (NPS) were performed in order to show the specificity of the assay (Supplementary Fig. 3). Among all the pro-inflammatory cytokines which were elevated by perfusion of the microchannels with NHS, IL-1β was reduced by treatment with DXS (p = 0.0095, Fig. 6) while C1 INH and APT070 did not show an effect. High levels of the soluble terminal complement complex sC5b-9 and C5a were found when cells were perfused with NHS alone (sC5b-9: 30547 ± 2932 ng/ml, C5a: 3298 ± 184.6 pg/ml), while addition of complement inhibitors significantly reduced both sC5b-9 and C5a generation [sC5b-9 (C1 INH: 19019 ± 10501 ng/ml, p = 0.004; APT070: 725 ± 585 ng/ml, p < 0.0001; DXS: 18605 ± 4181 ng/ml, C5a (C1 INH: 2123 ± 538 pg/ml, p = 0.002; APT070: 1543 ± 805.3 pg/ml, p < 0.0001; DXS: 808.4 ± 325.4 pg/ml, p < 0.0001; Fig. 7). Elevated levels of IL-1β and sC5b-9 as found in our *in vitro* system were also found in earlier *ex vivo* perfusion experiments performed with pig forelimbs^30^. We also found elevated levels of the growth factor bFGF in the perfusate when APT070 was used as compared to NHS alone (p < 0.05, Fig. 6). The significance of this finding is still unclear, also because APT070 has only rarely been used in xenotransplantation settings so far.

## Discussion

We have established an *in vitro* system for 3-dimensional growth of EC in microfluidic channels with circular cross sections under physiological flow conditions, mimicking small to medium sized arteries *in vivo*^31^. This microfluidic system was used to investigate endothelial cell activation in the context of a xenotransplantation setting.

Endothelial cells seeded into the microfluidic channels and grown under static conditions for the first two days aligned in the direction of flow as soon as exposure to shear stress was induced by pulsatile perfusion with cell culture medium. A frequent medium exchange after seeding the cells into the microchannels is required due to the high cell surface-to-volume ratio. After flow application, the EC monolayer covering the inner surface of the channels is continuously perfused with recirculating medium, reducing the need for medium exchange. In contrast to microchannels with a rectangular cross-section, the shear stress along the endothelial walls is homogeneous in our system and enables a better quantification of the effects of the flow on EC behaviour. Thanks to the transparency of the PDMS the system allows visualization as well as analysis of the microchannels by high resolution confocal microscopy. This is an advantage over *in vivo* systems and allows insights into molecular and cellular biological mechanisms which are not possible in animal models. Thanks to advanced settings of the confocal microscope we could show complete coverage of the inner surface of the round microchannels, both of 550 µm and 100 µm of diameter, by a confluent monolayer of EC, creating the impression of artificial small to medium sized arteries in a three-dimensional view. Furthermore, the closed system and recirculation of cell culture medium, with or without human serum or drugs, allows for the assessment of both acute and chronic effects on EC. Effector molecules in the fluid phase, which are an important way of communication between both adjacent and distant cells in the vasculature as well as in the blood stream, can develop their effects in the recirculatory microfluidic system and they can also be analyzed by ELISA or multi-plex assays.

After establishment of our *in vitro* model, we wanted to further validate the system by reproducing findings on complement activation as occurring *in vivo* in hyperacute or acute vascular rejection in xenotransplantation settings. A time- and volume-dependent increase of EC activation (E-selectin) and complement deposition (C3b/c) was observed. Indeed, the possibility to continuously perfuse the artificial blood vessels with high volumes of fresh serum as occurring *in vivo* is one of the main advantages of our system as compared for example to conventional 96-well plate assays. The results obtained from the testing of complement inhibitors – C1 INH, APT070 and DXS – regarding deposition of complement components and the prevention of EC activation revealed another important aspect of the new *in vitro* system, which is the possibility to assess the effects of different drugs in a purified system composed of an artificial endothelium exposed to pulsatile flow.

The reproducibility of the results is high as long as a confluent monolayer of cells is achieved and the cells are kept healthy before any kind of perfusion either with drugs or with serum.

A limitation of the current model is the use of serum to study activation of the EC growing on the inner surface of the microfluidic channels. *In vivo*, EC activation in transplantation, ischemia/reperfusion injury and other clinical settings occurs in the whole blood environment. Our study only includes the effect of complement and omits possible effects of the other plasma cascade systems, namely coagulation, fibrinolysis and kallikrein/kinin, as well as blood cells. Coating of the silicon tubings and connectors with heparin might allow the use of whole, non-anticoagulated blood for perfusion of the EC-microchannels and further improve the model. In the microchannels the ratio of EC-surface to blood volume is 73 cm^2^/ml for 550 µm diameter and 400 cm^2^/ml for 100 µm diameter, respectively. This would allow the exploitation of the natural, anticoagulant properties of EC when working with non-anticoagulated whole blood^10^. Modification of the model for use with whole, non-anticoagulated blood is currently under way.

## Methods

### Isolation and culture of porcine aortic endothelial cells

Porcine aortic endothelial cells (PAEC) were isolated from aortas by using a mechanical procedure. In brief, aortas were cut open and PAEC were isolated by gently rubbing the inner surface with a cotton wool bud. The cells were transferred into fibronectin-coated tissue culture flasks (Nalge Nunc International, Kamstrup, DK) and placed at 37 °C in a 5% CO_2_ incubator until confluence. DMEM cell culture medium (Thermo Fisher Scientific, Waltham, MA, USA) was used, supplemented with 10% heat-inactivated fetal bovine medium (FBS, Biochrom, Berlin, Germany), 100 IU/ml penicillin and 100 µg/ml streptomycin (Thermo Fisher Scientific), and 0.4% Endothelial Cell Growth Medium (ECGM) Supplement Mix (PromoCell, Heidelberg, Germany). Cells between passage 3 and 6 were used in the present study.

No animals were used specifically for the present study. Porcine aortas used for PAEC isolation were from animal experiments with pigs in the context of evaluation of surgical techniques and devices, as well as studies on xenotransplantation. All animal experiments were approved by the Veterinary Service of the Canton of Bern, Switzerland, and performed in accordance with national and international 3 R and ARRIVE guidelines^32^.

### Construction of microfluidic channels with round cross section

**Figure 8:**
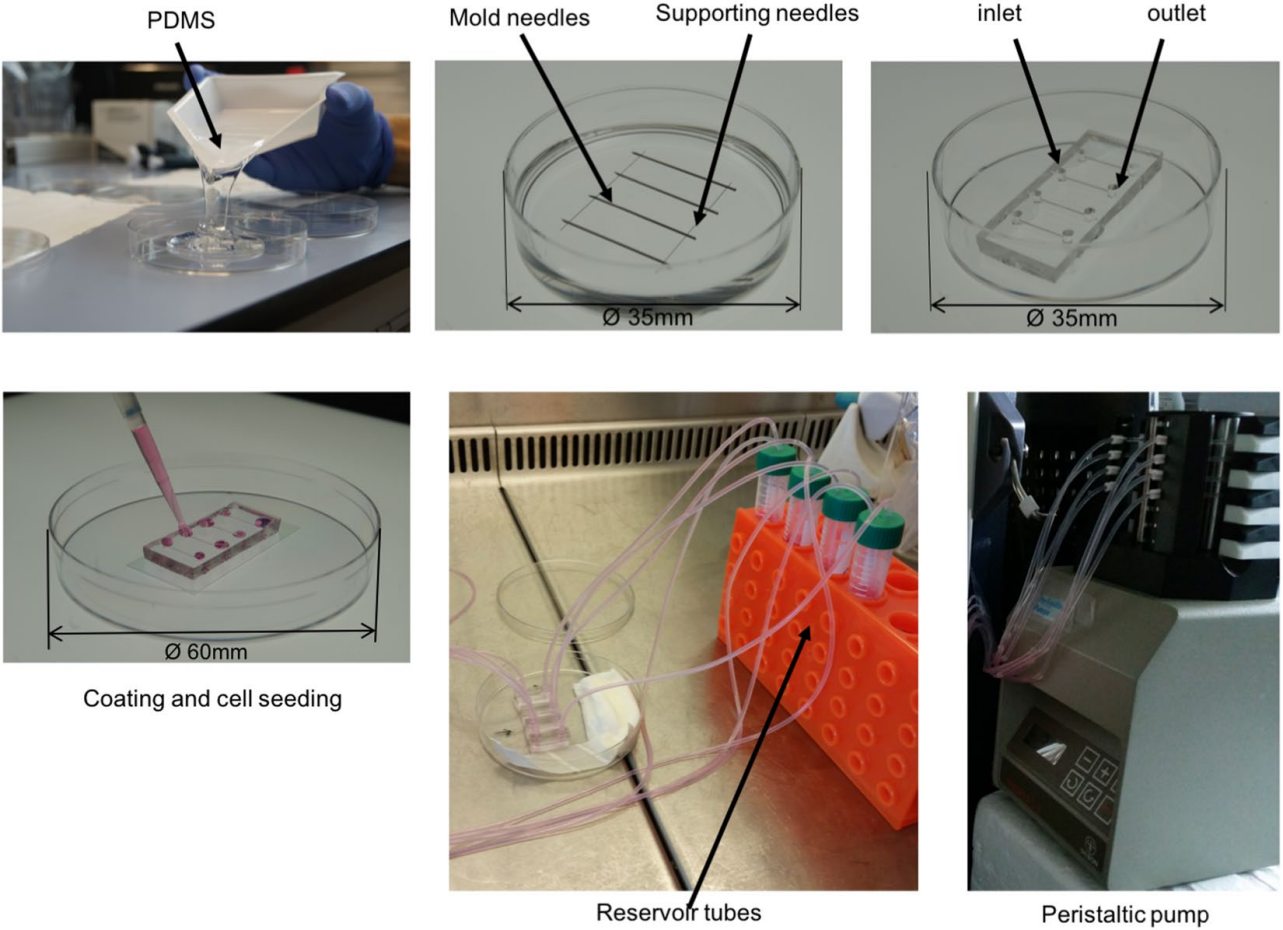
Schematic of microchannel fabrication and pump connecting. PDMS is poured into a Petri dish (Ø 60 mm). Supporting and mold needles are placed as shown in the picture and the whole Petri dish is incubated overnight at 60 °C. Needles are removed, inlet and outlet holes are made with a 2 mm biopsy puncher. Lateral holes are sealed with more PDMS. The second and the third steps show the device inside a Ø 35 mm Petri dish for a demonstration purpose. Normally everything is done using a Ø 60 mm Petri dish which can host up to 4 microchips. After plasma oxygen treatment, the microchip is bounded to a glass slide, coated with fibronectin and collagen I, and ultimately cells are seeded within the microchannels. One day after seeding a peristaltic pump is connected and a shear stress of 10 dyn/cm^2^ is applied.

Polydimethylsiloxane (PDMS, Sylgard 184, Dow Corning, Wiesbaden, Germany) was prepared by mixing 10 parts of elastomer silicone and 1 part of curing agent, and casted in a petri dish (Thermo Fisher Scientific). Sterile and pyrogen free needles with a diameter of 120 µm and a length of 3 cm (Seirin, Hamburg, MA, USA) were laid in parallel in the liquid uncured PDMS, at the bottom of the petri dish. Four mold needles of 550 µm or 100 µm diameter and 2.5 cm length (BD Biosciences, New Jersey, USA) were placed at a 90° angle on top of the thinner needles. The Luer connectors of the needles were cut off with a diagonal cutter before using the needles as molds. The PDMS with the needle-molds was cured at 60 °C overnight. PDMS chips were cut out, while needles were extracted horizontally. Inlet and outlet connectors to the microchannels were made with 2 mm biopsy punches (Shoney Scientific, Waukesha, USA). The hole, left from extraction of needles, between the edge of the PDMS gel and the inlet and outlet, respectively, was sealed with liquid PDMS and cured at 60 °C overnight. The final microfluidic chips contained four microchannels, mimicking small to medium sized arteries, with a diameter of 550 µm or 100 µm, respectively, and a length of 1 cm. The schematic for microchannel fabrication is shown in Fig. 8.

### Modification of PDMS surface in microchannels

Before seeding cells in the microfluidic channels, the inner surface of PDMS was modified to covalently bond extracellular matrix molecules^33^. Briefly, PDMS chips and standard glass slides were cleaned, activated in an oxygen plasma cleaner (Harrick Plasma, New York, USA) at 650 mTorr for 3 min, and bonded together. Immediately after bonding, the hydrophobic PDMS surface in the microchannels was silanized to make it hydrophilic by filling the channels with 5% 3-triethoxysilylpropylamine (APTES, Sigma-Aldrich, Buchs, Switzerland) and incubation for 20 min at room temperature. The channels were then washed with ultrapure water and treated with 0.1% glutaraldehyde (Sigma-Aldrich) for 30 min to provide a crosslinking substrate for the immobilization of extracellular matrix proteins. Microchannels were incubated with 50 µg/ml human fibronectin (Millipore, Schaffhausen, Switzerland) in PBS for 1 h at 37 °C or at room temperature overnight under UV light, followed by 100 μg/ml bovine collagen I in 0.2 mol/l acetic acid (Gibco, Thermo Fisher Scientific) at room temperature for 1.5 h. Cell culture medium containing 10% FBS was then rinsed through the microfluidic channels to block unspecific protein binding sites as well as to wash out unbound collagen I before cell loading.

### Cell loading and pulsatile flow

PAEC grown to confluence in T75 flasks were trypsinized with 0.05% EDTA-trypsin (Gibco, Thermo Fisher Scientific) and suspended in ECGM- and FBS-supplemented cell culture medium (DMEM) with 4% dextran from Leuconostoc spp. (Mw ~ 70,000, Sigma-Aldrich), to increase viscosity and promote cell adhesion. Cells at a density of 1 × 10^6^/ml were loaded into the microfluidic channels. The whole device was flipped upside down and placed in an incubator at 37 °C/5% CO_2_ for 10–15 minutes to promote cell adhesion on the upper part of the microchannel. Subsequently cell attachment was checked under the microscope and if necessary more cells were added and the unflipped device placed back in the incubator. Cells were then cultured under static conditions with 2–3 cell culture medium changes at intervals of 2 h, followed by overnight incubation. Growth of the cells was checked daily under an inverted microscope. After confluency was reached, a peristaltic pump – Minipuls 3 with 8 channels (Gilson, Villiers le bel, France) – was connected to the microfluidic channels via sterile silicon tubing with stoppers (Gilson) and extension silicon tubings (Gobatec, Bern, Switzerland). These tubes were autoclaved and extensively flushed with distilled water and PBS, followed by cell culture medium with 4% dextran. A medium reservoir in a 15 ml sterile tube (Corning, Berlin, Germany) was connected to each microchannel and placed in the 37 °C incubator together with the microfluidic device. PAEC in the microfluidic channels were maintained in DMEM under pulsatile flow, starting overnight with a low shear stress of 0.04 dyn/cm^2^, corresponding to 0.5 pump head rotations per minute, equaling 5 beats per minute (bpm) because of the presence of 10 rollers on the pump head. Thereafter the shear stress and pulse rate was gradually increased by 10 bpm per hour, until the desired shear stress of 10 dyn/cm^2^ at 70 bpm was reached. This shear stress of 10 dyn/cm^2^, corresponding to a flow of 600 µl per minute for 550 µm channels, was maintained for two days in the present study. Calculations of the shear stress were performed based on the equation (1):

$${\rm{SS}}=4\mu Q/{{\rm{\pi }}}^{\ast }{{\rm{R}}}^{3}$$

where µ is the viscosity of the medium, Q is the flow rate and R represents the radius of the microchannel. The system can be maintained for at least 7 days with exchange of the medium every 2–3 days. Cell morphology was assessed under a bright field microscope (DMI 4000B, Leica Microsystems Schweiz, Heerbrugg, Switzerland).

### Human serum preparation

Human blood was drawn from healthy volunteers into polypropylene tubes containing glass beads (S-Monovette, Sarstedt, Germany) and allowed to clot for 30 min at room temperature. The clot was removed by centrifugation for 10 min at 2000 × g in a refrigerated centrifuge (4 °C) and the supernatant collected and stored at −80 °C. In the present study sera from different donors with different blood groups were used, mostly as pool of at least 3 donors. Details are given in the respective figure legends. All experimental protocols were reviewed and approved by the University of Bern and carried out in accordance with the University of Bern regulations. All human blood samples were obtained with informed consent according to Swiss jurisdiction and ethics guidelines of the Bern University Hospital.

### Perfusion of PAEC with normal human serum and complement inhibitors

After two days of pulsatile flow, cell culture medium was replaced with normal human serum (NHS) 1:10 diluted in 4% dextran DMEM without supplements. PAEC were perfused for different periods of time (10 min, 30 min, 60 min, 120 min). The perfusate (1:10 diluted NHS in 4% dextran DMEM with or without complement inhibitors) was present in 15 ml reservoir tubes (Nalge NUNC) and perfusion was performed in a closed circuit so that the perfusate was recirculated. Usually 10 ml of perfusate were used, but for some experiments the amount was varied from 3 to 10 ml, with a control of static incubation with 200 µl. Four groups were made: Group 1: NHS alone, Group 2: NHS + 10 IU/ml C1 inhibitor (C1 INH, Berinert, provided by CSL Behring, Marburg, Germany), Group 3: NHS + 0.25 mg/ml APT070 (a recombinant, membrane-targeted complement inhibitor based on complement receptor 1, provided by Richard Smith, King’s College, London, UK), Group 4: NHS + 0.3 mg/ml low molecular weight dextran sulfate (DXS, Mw ~5000, provided by Tikomed, Viken, Sweden). For each group, experiments with 3–5 channels were performed. Finally, perfusate was collected and stored at −80 °C. EC in the microchannels were used for immunofluorescence staining.

### Immunofluorescence staining

Immunofluorescence staining was performed to assess the establishment of a confluent EC monolayer on the inner surface of the microchannels, to characterize endothelial cells and to assess deposition of complement components as well as EC activation. In brief, cells in the microfluidic channels were washed with PBS, fixed with 4% formaldehyde for 15 min, and blocked with PBS-3% BSA for 45 min. Incubation with primary antibodies was done at 4 °C overnight, followed by secondary antibodies and 4**’**,6-diamidino-2-phenylindole (DAPI). The primary antibodies used were: rat anti-porcine CD31 (mAB33871, R&D, Minneapolis, USA), goat anti-human VE-cadherin (sc-6458, Santa Cruz, Texas, USA), rabbit anti-human von Willebrand factor (vWF, A0082, Dako, Glostrup, Denmark), rabbit anti-human C3b/c-FITC (F0201, Dako), rabbit anti-human C4b/c-FITC (F0169, Dako), Goat anti-human C6 (A307, Quidel, San Diego, USA), mouse anti-human E-selectin (S-9555, Sigma-Aldrich). The secondary antibodies were goat anti-rat IgG Cy3 (112-166-003, Jackson ImmunoResearch, West Grove, PA, USA), donkey anti-goat alexa488 (A21082, Thermo Fisher Scientific), sheep anti-rabbit IgG Cy3 (C2306, Sigma-Aldrich), donkey anti-goat IgG alexa488 (A11055, Thermo Fisher Scientific, MA, USA), goat anti-mouse IgM FITC (115-097-020; Jackson ImmunoResearch), goat anti-mouse IgG alexa488 (A21121, Thermo Fisher Scientific). Nuclei were stained with DAPI (Boehringer, Roche Diagnostics, Indianapolis, IN, USA). In addition, cytoskeleton filamentous actin (F-actin) was stained with Rhodamine Phalloidin (PHDR1, Cytoskeleton, Inc., Denver, USA). Images were taken at 10x and 63x with a confocal laser-scanning microscope (LSM 710, Zeiss, Feldbach, Switzerland) and analyzed by ImageJ (National Institutes of Health, Bethesda, MD, USA). The thickness of the entire microfluidic device is 0.5 cm and the distance between the bottom of the device and the bottom of the microchannel is 120 µm which allows a good imaging. In addition, z-stack images were processed by Imaris 8.2 software (Bitplane, Zurich, Switzerland).

### Quantification of cellular alignment

To quantify cellular alignment with the direction of flow, cell orientation was analyzed and quantified using the FibrilTool plugin function in Fiji (http://fiji.sc/Fiji) following the published protocol^34^ both under static and flow conditions. Fluorescent signals from CD31 and F-actin staining were used. Three images per channel were analyzed to obtain the mean fluorescence intensity.

### Detection of porcine cytokines and complement activation markers by Bio-Plex/ELISA

To determine the concentrations of porcine-specific cytokines [interleukin (IL)-6, IL-1β, IL-10, tumor necrosis factor alpha (TNF-α)], basic fibroblast growth factor (bFGF), as well as the complement activation marker soluble (s)C5b-9 in perfusate samples, a multiplex xMAP technology (Luminex) assay was performed according to a custom-made protocol developed by our group^35^. In brief, microbeads (Luminex) were coupled with respective capture antibodies using the Bio-Plex amine coupling kit (Bio-Rad). Coupled beads were then incubated with samples, followed by biotinylated detection antibodies and Streptavidin-R-PE (922721, Qiagen, Hilden, Germany). Measurement and data analysis were performed with a Flexmap 3D reader and the Bio-Plex Manager software version 6.1 (Bio-Rad). Concentrations of human C5a were detected by ELISA using a commercially available kit (DuoSet, R&D Systems, Minneapolis, USA).

### Statistical analysis

All data are presented as mean ± standard deviation (SD). Statistical analyses were performed by GraphPad Prism 6 software (GraphPad, San Diego, CA, USA) using one-way analysis of variance (ANOVA) followed by Fisher’s LSD post hoc test to compare means of all groups. For comparison of cell orientation, Mann-Whitney U test was used. P values < 0.05 were considered statistically significant.

### Data availability

The complete data sets of this article are available upon request.

## Electronic supplementary material

Dataset 1

Supplementary video 1
