# Supplementary material for: 3D artificial round section micro-vessels to investigate endothelial cells under physiological flow conditions
Source: Sci Rep. 2018 Apr 12;8:5898. doi: 10.1038/s41598-018-24273-7 (PMC5897395; doi:10.1038/s41598-018-24273-7)
Supplement: Supplementary file 1 — Dataset 1 [file 41598_2018_24273_MOESM1_ESM.docx]

**3D artificial round section micro-vessels to investigate endothelial cells under physiological flow conditions**

Riccardo Sfriso^ab†^, Shengye Zhang^abc†^, Colette Andrea Bichsel^d^, Oliver Steck^a^, Alain Despont^a^, Olivier Thierry Guenat^e^, Robert Rieben^a*^

^a^Department for Biomedical Research, University of Bern, Switzerland

^b^Graduate School for Cellular and Biomedical Sciences, University of Bern, Switzerland.

^c^First Affiliated Hospital of Zhengzhou University, Zhengzhou, China

^d^Vascular Biology Program, Boston Children’s Hospital and Harvard Medical School, Boston MA, USA

^e^ARTORG Center for Biomedical Engineering Research, University of Bern, Switzerland

^†^Riccardo Sfriso and Shengye Zhang equally contributed to this publication

^*^Correspondence and requests for material should be addressed to R.R. ([robert.rieben@dbmr.unibe.ch](mailto:robert.rieben@dbmr.unibe.ch))

**Supplementary Figure 1. Antibody binding to fibronectin/collagen-coated microchannels.** Microchannels were prepared as described in the methods except the cell seeding. Fibronectin/collagen-coated microchannels were perfused for 2 hours with 1:10 diluted NHS and assessment of human IgG (a) and IgM (b) binding was done by immunofluorescence staining and confocal microscopy. The confocal images show the absence of both IgG and IgM binding (green staining) on fibronectin/collagen coated microchannels while a significant antibody binding was observed when wildtype (WT) cells were perfused with NHS. Shown are mean values ± SD with indication of statistically significant differences between the groups, n=5, p-value: ** p<0.01, *** p=0.0001, **** p<0.0001). Scale bar represents 100 µm.

**Supplementary Figure 2. Allogeneic perfusion of PAEC and HAEC.** Both PAEC and HAEC were perfused with 1:10 diluted NPS and 1:10 diluted NHS respectively for 120 min. Immunofluorescence staining for C3b/c was performed. Confocal pictures were acquired and the fluorescence intensity was quantified by using ImageJ software. Bars show mean values ± SD with indication of statistically significant differences between the groups, PAEC n=10, PAEC+NPS n=5, PAEC+NHS n=10, HAEC no NHS n=1, HAEC+NHS n=3, p-value: **** p<0.0001).

**Supplementary Figure 3. Analysis of cytokines/growth factors on pre-perfusion NHS and normal pig serum (NPS) as well as on perfusate.** 1:10 diluted NHS and NPS were analyzed both before and after perfusion through EC-coated microchannels. Values of NHS pre-perfusion and NHS after perfusion are taken from Fig.6. Analysis of the cell culture medium after perfusion was also performed as control. Shown are mean values ± SD with indication of statistically significant differences between the groups, n=2, NHS post-perfusion n=5, p-value: * p=0.01, ** p<0.01, *** p=0.0001).

**Supplementary Figure 4. Analysis of soluble complement (C5a and sC5b-9) on pre-perfusion NHS and normal pig serum (NPS) as well as on perfusate.** 1:10 diluted NHS and NPS were analyzed both before and after perfusion through EC-coated microchannels. Values of NHS pre-perfusion and NHS after perfusion are taken from Fig.7. Analysis of the cell culture medium after perfusion was also performed as control. Shown are mean values ± SD with indication of statistically significant differences between the groups, n=2, NHS post-perfusion n=5, p-value: **** p<0.0001).
